# Second-Line Treatment of NSCLC—The Pan-ErbB Inhibitor Afatinib in Times of Shifting Paradigms

**DOI:** 10.3389/fmed.2017.00009

**Published:** 2017-02-13

**Authors:** Jens Köhler

**Affiliations:** ^1^Department of Medical Oncology, Dana-Farber Cancer Institute, Harvard Medical School, Boston, MA, USA; ^2^West German Cancer Center Essen, Department of Medical Oncology, Division of Thoracic Oncology, University Hospital Essen, Essen, North-Rhine Westphalia, Germany

**Keywords:** afatinib, EGFR mutation, TKI, second-line treatment, NSCLC, squamous cell carcinoma, T790M-specific inhibitors, checkpoint blockade

## Abstract

In contrast to the established role of epidermal growth factor receptor (EGFR) inhibitors for the first-line treatment of patients with non-small cell lung cancer (NSCLC) harboring activating *EGFR* mutations, the role of EGFR blockade and of *EGFR* molecular testing in the second-line treatment remains less clear. The irreversible pan-ErbB family inhibitor afatinib (Gi(l)otrif^®^) was recently FDA- and EMA-approved for the second-line treatment of NSCLC with squamous cell histology irrespective of the *EGFR* mutational status (LUX-Lung 8). Contrariwise, results from the TAILOR and DELTA trials among retrospective biomarker analyses show the predictive value of the *EGFR* mutational status for efficacy of reversible EGFR inhibitors also as a second-line therapy. This mini review critically summarizes the current role of EGFR-targeting strategies in the second-line treatment of NSCLC with special respect to afatinib in light of emerging *T790M*-specific EGFR and immune check point inhibitors. The review also emphasizes the urgent need for reliable biomarkers to guide therapeutic decision-making and outlines prospective changes to the second-line landscape with some of the current second-line treatment concepts likely to be moved to the first-line.

## Introduction

Over the past decade, various genomic alterations relevant for non-small cell lung cancer (NSCLC) biology (“oncogene addiction”) were discovered and have subsequently changed the treatment paradigm from a histology-oriented to a biomarker-driven approach [reviewed by Thomas et al. (1)]. Historically, docetaxel was the gold standard second-line treatment (2) until erlotinib (Tarceva^®^), a first-generation tyrosine kinase inhibitor (TKI) of the epidermal growth factor receptor (EGFR) was FDA-approved in 2004 as maintenance therapy and for second and subsequent line treatment, after failure of chemotherapy in unselected patients (3). In the meantime, several phase-III trials compared EGFR TKIs with chemotherapy and have established EGFR TKIs as the standard first-line treatment for patients with *EGFR*-mutant NSCLC (4–7). Nowadays, not less than three EGFR TKIs—erlotinib, gefitinib (Iressa^®^) and the pan-ErbB family inhibitor afatinib (Giotrif^®^)—are licensed for the first-line treatment. Drug reimbursement is bound to the presence of a common activating *EGFR* mutation (i.e., exon 19 deletions and L858R point mutations) detected by FDA-approved tests [erlotinib—cobas^®^; gefitinib (Iressa^®^) and afatinib (Gi(l)otrif^®^)—therascreen EGFR RGQ]. However, the relevance of *EGFR* mutations for the second-line decision-making process remained less clear, and erlotinib (for all NSCLC) as well as afatinib (for squamous cell histology only) have initially been FDA-approved irrespective of *EGFR* mutational status or other predictive markers (3, 8). Several recent prospective clinical trials (TAILOR, DELTA) and retrospective biomarker analyses challenge this broad approval and emphasize the need for *EGFR* mutational re-testing ahead of the second-line therapy if not performed at diagnosis (9, 10).

## Evidence for Clinical Efficacy of EGFR TKIs in the Second Line

The use of first-generation EGFR TKIs like erlotinib and gefitinib in the second-line treatment of patients with *EGFR*-mutant NSCLC is supported by prospective single-arm studies, retrospective biomarker analyses of phase-II studies and subgroup analyses from phase-III studies (11–20). Several randomized trials have compared single-agent EGFR TKIs with single-agent chemotherapy and showed an improvement in progression-free survival (PFS) but mostly not in overall survival (OS) with chemotherapy compared with EGFR TKIs in an *EGFR* wild-type population (9, 10, 19, 21–28). Afatinib has been tested in the worldwide LUX-Lung trial program and second-line studies included LUX-Lung 2 (first- or second-line, single-arm), LUX-Lung 4 (second-line or beyond), and the head-to-head comparison with erlotinib in LUX-Lung 8 (second-line) (8, 29). Afatinib like dacomitinib (the latter is not FDA-approved yet) irreversibly inhibits all ErbB family members and was supposed to overcome resistance mediated by secondary *EGFR T790M* mutations (30) which occur in ~50–60% of cases upon progression with reversible EGFR TKIs (31). Both drugs demonstrated promising activity against T790M in preclinical models but failed to overcome T790M-mediated resistance in patients due to dose-limiting toxicity resulting from inhibition of wild-type EGFR (32). Furthermore, analyses of small numbers of re-biopsy samples suggest that treatment with afatinib in the first-line results in similar rates (~50–60%) of secondary *T790M* mutations upon progression compared to reversible EGFR TKIs (31, 33). This may be due to the high frequency (up to 80%) of pretreatment *EGFR T790M* mutations (34). However, the results from LUX-Lung 4 and 5 suggested that some patients not only may benefit from afatinib after acquired resistance to gefitinib/erlotinib but also from continued ErbB inhibition during chemotherapy versus switching to single-agent chemotherapy after progression with EGFR TKIs (35). The LUX-Lung 5 results have yet not let to changes in second-line treatment recommendations in terms of combining EGFR inhibition with cytotoxic chemotherapy post-progression in patients with *EGFR*-mutant NSCLC who initially responded to EGFR TKI treatment.

## Afatinib in NSCLC with Squamous Cell Histology

Currently, treatment paradigms are most dramatically changing in tumors with squamous cell histology. This entity has unmet medical needs even though the incidence in Western countries is decreasing (25% of all lung cancer cases). Reflecting the tobacco carcinogenesis, tumors are genomically complex yet *EGFR* mutations are sporadic, and *EGFR* molecular testing is not routinely performed in this subgroup (36). Molecular analyses indicated that pan-ErbB blockade could be of therapeutic benefit in squamous cell tumors due to multiple genetic aberrations in ErbB receptors (*HER2*: 4%, *HER3*: 2%) and in downstream signaling molecules (*KRAS*: 3%, *HRAS*: 3%, *BRAF*: 4%, *NF1*: 11%, *NRG1*) (36). Furthermore, 20–30% of tumors overexpress HER2 and HER3. Whereas erlotinib was the only approved second-line TKI in squamous cell lung cancer since 2004, afatinib received FDA- and EMA-approval for the second-line treatment of squamous cell NSCLC in 2016 based on results of the head-to-head (against erlotinib) study LUX-Lung 8 (8). This approval is irrespective of the intratumoral *EGFR* mutational status. Supposedly, the improved OS [median 7.9 months (95% CI 7.2–8.7) versus 6.8 months (5.9–7.8); HR 0.81 (95% CI 0.69–0.95), *p* = 0.0077] is unlikely driven by the inhibition of mutant *EGFR* which was found in only 6% of the patients but rather by the broader irreversible pan-ErbB blockade with afatinib compared to erlotinib.

## Newly Emerging Third-Generation EGFR Inhibitors and Immune Checkpoint Blockade in the Second-Line Treatment

After second-generation EGFR TKIs failed to effectively overcome T790M-mediated resistance in the clinical setting, drugs that specifically inhibit EGFR T790M without affecting wild-type EGFR were developed subsequently. Osimertinib (Tagrisso^®^), a EGFR T790M-specific kinase inhibitor, inhibits EGFR exon 18, 19, and 21 mutations and the drug-resistant *T790M* mutation and received accelerated FDA approval in 2015. Response rates to osimertinib in patients with *T790M*-positive tumors after first-generation EGFR TKI are comparable to those with first-line EGFR TKI (58–61%) and the median PFS reached 9.6 months compared to 2.8 months in EGFR T790M-negative patients. Osimertinib has a better toxicity profile than first- and second-generation EGFR TKI due to the reduced wild-type EGFR inhibition. Common adverse events are class-specific (i.e., diarrhea, rash, nail toxicity) but were generally mild to moderate (37).

Other promising therapeutic concepts that experienced a tremendous renaissance especially in squamous cell NSCLC include the modulation of the tumor vasculature [anti-VEGFR-2 antibody ramucirumab (Cyramza^®^), REVEL trial] (38) and of the immune environment. The latter strategy enhances the patient’s natural immune response to cancer mainly *via* CD8+ cells. Cytotoxic T-lymphocyte-associated protein-4 (CTLA-4) and programmed cell death protein (PD-1) have been identified as important targets which are expressed on activated T cells and interact with ligands on antigen-presenting cells thereby limiting the immune response. Both, the anti-PD-1 monoclonal antibody Nivolumab (Opdivo^®^) (39, 40) and pembrolizumab (Keytruda^®^) (41) have been FDA-approved for PD-L1-positive (defined as a tumor proportion score ≥ 50%) metastatic squamous (nivolumab) or squamous and non-squamous (pembrolizumab) NSCLC lacking *EGFR* or *ALK* mutations with progression to platinum-based chemotherapy. Other antibodies targeting PD-L1 like atezolizumab (MPDL3280A) confirm the efficacy of this innovative concept of immune checkpoint blockade (42).

## Conclusion and Outlook

Compared to the their role in the first-line, reversible (erlotinib, gefitinib) and irreversible (afatinib) EGFR TKIs have relatively less impact on the second-line treatment of patients with advanced NSCLC. Afatinib, however, was recently approved for patients with squamous cell NSCLC irrespective of the *EGFR* mutational status. With the advent of innovative treatment concepts as e.g., immune checkpoint blockade or T790M-specific EGFR inhibition, it is likely that EGFR TKIs will be further pushed into the first-line where they already today face ongoing head-to-head comparisons with the EGFR T790M-specific inhibitor osimertinib to identify the most effective upfront treatment option for patients with *EGFR*-mutant NSCLC (e.g., FLAURA trial: osimertinib versus gefitinib or erlotinib).

Currently, patients with *EGFR*-mutant tumors should be treated with EGFR TKIs as soon as possible, ideally in the first-line setting. This is supported by several first-line phase-III clinical trials, which showed higher response rates (>70%) (5, 43) as if the EGFR TKI was given in the second-line (27–67.4%) even though some of the reported data on response rates have been conflicting (5, 18, 19, 43, 44). Apart from the pooled LUX-Lung 3 and 6 analyses, all EGFR TKI first-line trials failed to show an OS benefit (45). This is likely confounded by crossover of patients to EGFR TKI post-progression to first-line chemotherapy. From the only prospective randomized TORCH trial which compared first-line EGFR TKI followed by chemotherapy with first-line chemotherapy followed by second-line EGFR TKI, the authors concluded, that patients with *EGFR* mutations would experience greater benefit from first-line EGFR TKI followed by second-line chemotherapy. However, patients in this study were not selected by *EGFR* mutational status (only 14.2% were *EGFR* mutation positive) and the small sample size as well as the fact that only 60% of patients in both arms received second-line treatment furthermore confounded the result (46). Numerous arguments yet support the application of EGFR TKI in the first-line over second-line: quality of life during EGFR TKI treatment is better compared to first-line chemotherapy especially in patients with poor performance status, whole-brain irradiation with its detrimental consequences on cognitive functions for patients with brain metastasis may be delayed by EGFR TKIs (47–49) and giving EGFR TKIs upfront increases the chance of TKI exposure for those patients whose tumors harbor the target. This is supported by the fact that about 1/3 of patients with *EGFR* mutations assigned to first-line chemotherapy did not receive EGFR TKI as salvage therapy in IPASS, WJTOG 3405, and OPTIMAL (4, 6, 50). LUX-Lung 6 reported the longest PFS (13.7 months) of all first-line EGFR TKIs and two head-to-head comparison studies [LUX-Lung 7 (first-line) and 8 (second-line)] were slightly in favor of afatinib over erlotinib and gefitinib even though there was some criticism about the interpretation of results and the publication strategy (26). Nevertheless, these trials indicate that afatinib is a highly effective drug in this setting but comes with numerically higher side effect rates compared to erlotinib and gefitinib (8, 51, 52). These toxicities are effectively manageable by supportive measures (53, 54) and tolerability-guided dose reductions which do not affect therapeutic efficacy (55). Especially afatinib, however, will be confronted with EGFR T790M-specific inhibitors like osimertinib in the first-line setting as the latter have a more favorable toxicity profile due to less wild-type EGFR inhibition.

If *EGFR* mutational testing has not been performed ahead of the first-line therapy—it is estimated that 15 to 35% of patients have insufficient tumor tissue for genotyping (56, 57), patients should be considered for repeated testing before starting second-line therapy. Plasma-genotyping, a technique that uses cell-free (cf)DNA, may be an important alternative to the classical biopsy approach in this scenario (58, 59) and it is highly likely that “liquid biopsies” will become available for many known oncogenic and resistance mutations in the near future. This may substantially change the decision-making process as liquid biopsies will enable the physician to monitor development of resistance more promptly and to decide more accurately on therapeutic consequences (60). TAILOR, DELTA, and other trials indicate the predictive value of *EGFR* mutational status on EGFR TKIs in the second-line (9, 10). In particular, the TAILOR study clearly suggests that second-line docetaxel is superior to erlotinib in terms of survival in all patients with *EGFR* wild-type NSCLC who are able to tolerate toxicities of chemotherapy. DELTA and other trials (CTONG0806 (28) and NCT01783834 (61): pemetrexed versus gefitinib) as well as a meta-analysis by Li et al. (62) point into the same direction with a better PFS for second-line chemotherapy in *EGFR* wild-type patients. On the contrary, there is evidence to suggest that patients with *EGFR*-mutant NSCLC who are still TKI naive perform better with EGFR TKIs (9, 10, 19, 21–28). In this context, reacting to the JUNO trial results (not fully published yet), FDA restricted the indication for erlotinib as maintenance or second or greater line treatment to those NSCLC patients whose tumors harbor common *EGFR* mutations in October 2016.

If the *EGFR* mutation status remains unknown for the second-line treatment decision, a preferred strategy would be to offer nivolumab for squamous NSCLC or pembrolizumab for squamous and non-squamous histology (after platinum-based chemotherapy if PD-L1 expression ≥ 50%). The approval of immune checkpoint inhibitors will consequently push docetaxel—long the standard of care treatment in the second-line—to the third-line or even beyond. Especially for squamous cell NSCLC, based on the positive survival results of the SQUIRE study which tested the human EGFR monoclonal antibody necitumumab in combination with cisplatin-gemcitabine chemotherapy, the treatment might soon change even in the first-line setting (63). Big efforts are furthermore ongoing to advance biomarker-driven therapies for patients with squamous cell carcinoma of the lung within the Lung-MAP studies (64) and it is also not a far-fetched vision that immune checkpoint inhibitors will have a role in untreated advanced lung cancer. Currently, more than 10 randomized trials (among them KEYNOTE, CHECKMATE, IMPOWER) are ongoing and the question will rather be how checkpoint inhibitors will integrate into the upfront setting, as monotherapy or in concurrent or sequential combination with chemotherapy.

Other important questions remain that may open up new indications for afatinib, but also other EGFR TKIs as to which drug is most effective in controlling brain metastases and rare *EGFR* mutations. It is known, that patients with *EGFR* mutations have an increased risk especially for leptomeningeal tumor dissemination (65, 66). Penetration of the blood-brain barrier as well as clinical efficacy have been described for both afatinib (47–49) and osimertinib (67). Other EGFR inhibitors with high *in vivo* CNS penetration (e.g., AZD3759) are currently under early clinical phase evaluation. To determine the most effective drug for CNS disease, also more systematic investigation of the mutational spectrum in brain metastases is required. In this context, surprisingly, a restrospective study found the majority of CNS and leptomeningeal metastases to be negative for EGFR T790M despite of T790M positivity in the extracranial tumor (spatiotemporal heterogeneity) (68). This may argue against T790M-specific and rather for first- or second-generation EGFR TKIs.

Another field of current interest are less common *EGFR* mutations which together represent about 10% of all *EGFR* mutations (69). Especially afatinib may be a good option for these rare *EGFR* mutations that include exon 18-21 duplications, G719X, Del18, E709K, insertions in exon 19, S768I, or L861Q as erlotinib, osimertinib and gefitinib showed only moderate activities in these mutations (70, 71, 72). Osimertinib contrariwise may be effective in rare exon 20 insertions whereas nazartinib (EGF816) shows promising efficacy in the majority of exon 20 mutations. The quinazoline-based EGFR inhibitors, gefitinib and afatinib finally proofed efficacy in tumors containing a common *EGFR* mutation (i.e., Del19 or L858R), in conjunction with L718Q, L844V, or C797S (73, 74).

To summarize, treatment paradigms for NSCLC patients in the second-line are currently experiencing dramatic changes. Many of the currently tested innovative concepts will likely move forward to the first-line treatment, whereas other strategies and possibly indications for EGFR TKIs (as, e.g., continued ErbB blockade post-progression, TKI-specific efficacy in rare mutations) may be established in the second-line. One necessity that all therapeutic concepts and treatment lines share in common is the urgent need for reliable predictive factors in times of increasing treatment costs. These are still not available for anti-angiogenic agents like ramucirumab and it remains unclear, if any predictive biomarker will help to select patients with squamous cell NSCLC for afatinib treatment in the future.

## Author Contributions

JK wrote the manuscript and is responsible for all its content.

## Conflict of Interest Statement

JK received consultant honoraria from Boehringer Ingelheim for writing and publishing two reviews on afatinib unrelated to this article and travel grants from Roche, Amgen, and Lilly. He currently is a Mildred-Scheel scholar of the German Cancer Aid Foundation (Deutsche Krebshilfe).
